# Lifestyle factors that affect cognitive function–a longitudinal objective analysis

**DOI:** 10.3389/fpubh.2023.1215419

**Published:** 2023-07-31

**Authors:** Noriyuki Kimura, Yuuki Sasaki, Teruaki Masuda, Takuya Ataka, Atsuko Eguchi, Tatsuyuki Kakuma, Etsuro Matsubara

**Affiliations:** ^1^Department of Neurology, Faculty of Medicine, Oita University, Yufu, Japan; ^2^Biostatics Center, Kurume University, Kurume, Japan

**Keywords:** prospective cohort study, longitudinal analysis, physical activity, sleep quality, cognitive decline

## Abstract

**Background:**

Identifying lifestyle factors associated with cognitive decline has critical clinical and public health implications for dementia prevention in later life. The longitudinal associations of sleep and physical activity with cognitive function remain unclear. This study examined whether objectively measured sleep and physical activity were longitudinally associated with cognitive function in older adults over a three-year period.

**Methods:**

This prospective cohort study enrolled 855 community-dwelling adults aged 65 and older, who were followed from 2015 to 2019. All participants were required to wear a wearable sensor for 7 consecutive days every 3 months and had annual cognitive assessments. Wearable sensor data (August 2015–September 2019) and Mini-Mental State Examination (MMSE) scores (August 2015–April 2019) were collected over 3 years of follow-up. First, principal component analysis was conducted to reduce the dimensions of the sleep and physical activity variables to two principal components for inclusion in a mixed-effects model. The sleep index consisted of sleep efficiency, time awake after sleep onset, and waking frequency. The physical activity index was composed of walking comprised steps per day and time devoted to light or moderate-to-vigorous physical activity. A higher sleep index indicated poor sleep quality, whereas a lower physical activity index indicated less physical activity. Second, a linear mixed effect model was used to examine the longitudinal association of sleep and physical activity indices with cognitive decline over time.

**Results:**

In total, 855 adults were recruited for this study at baseline. Of these, 729 adults (85.3%) completed a measurement of lifestyle factors and an annual cognitive testing, whereas 126 were excluded because of death or loss during follow-up. After adjusting for age, sex, education level, and time, the sleep index was inversely associated with MMSE scores (estimate, −0.06229; standard error, 0.02202; *p* = 0.0047) and the physical activity index was positively associated with MMSE scores (estimate, 0.06699; standard error, 0.03343; *p* = 0.0453).

**Conclusion:**

Poor sleep quality and lower physical activity were significant risk factors for subsequent cognitive decline in older adults. The present study facilitates the development of novel evidence-based interventions for physical activity and sleep quality to delay cognitive decline.

## Introduction

Dementia is a major public health issue globally because of population aging. The prevalence of dementia among people aged 60 years and older in Japan was approximately 5.1 million in 2016, and if current trends are maintained, it is estimated that 5.03 million people will develop dementia in 2025 (1). Therefore, identifying modifiable risk factors is critical for providing information about public health strategies for dementia prevention and improving patient quality of life. Cohort studies indicated that lower levels of education, vascular risk factors, unhealthy lifestyles, and hearing loss have adverse effects on cognitive function (2, 3). Sleep disturbance and physical inactivity, in particular, are major concerns in an aging society and important risk factors for dementia or Alzheimer’s disease (AD) later in life (2–5). The majority of previous cohort studies have examined the association between self-reported sleep or physical activity at baseline and subsequent cognitive decline or incidence dementia (6, 7). Subjective self-report questionnaires, however, have poor reliability and consistency due to recall bias or misclassification (8, 9), and are limited to capture nonexercise physical activity that accounts for most of the energy expenditure among older adults. Recently, wearable sensors have been used to evaluate lifestyle factors in large epidemiological studies because they provide noninvasive, cost-effective tools for the objective and continuous measurement of total daily physical activity and sleep patterns without recall bias. Moreover, longitudinal studies are valuable in determining the prospective association between habitual sleep or physical activity and the risk of subsequent cognitive decline or development of dementia (10–18). In fact, previous studies linking objective sleep with cognitive function showed the association of lower sleep efficiency, longer sleep latency, and sleep fragmentation, such as greater wakefulness and number of long wake episodes, were subsequent cognitive decline the risk of developing AD (10–13) and those linking objective physical activity with cognitive function showed the association of total physical activity with lower risk of cognitive decline (14–18). However, these studies objectively measured sleep and physical activity using wearable sensor only at baseline. Since the sleep–wake cycle, physical activity, and Mental State Examination (MMSE) scores change with aging (19–21), periodic measurement of sleep, physical activity, and cognitive function over follow-up period is helpful in understanding their dynamic association. Although several studies have examined the longitudinal association between change in self-reported sleep or physical activity and subsequent cognitive decline over time (22–27), to the best of our knowledge, few studies have focused on the longitudinal associations of objectively measured sleep and physical activity with cognitive function. Therefore, we conducted a prospective cohort study to determine the longitudinal association of objectively measured lifestyle factors with MMSE scores in community-dwelling older adults from 2015 to 2019. The aim of this study was to determine whether lifestyle factors were longitudinally associated with cognitive decline over a three-year period. We hypothesized that poor sleep quality or low physical activity would be associated with cognitive decline. The present study may improve the understanding of the influence of modifiable lifestyle factors on cognitive function in later life.

## Methods

### Study participants

**Figure 1:**
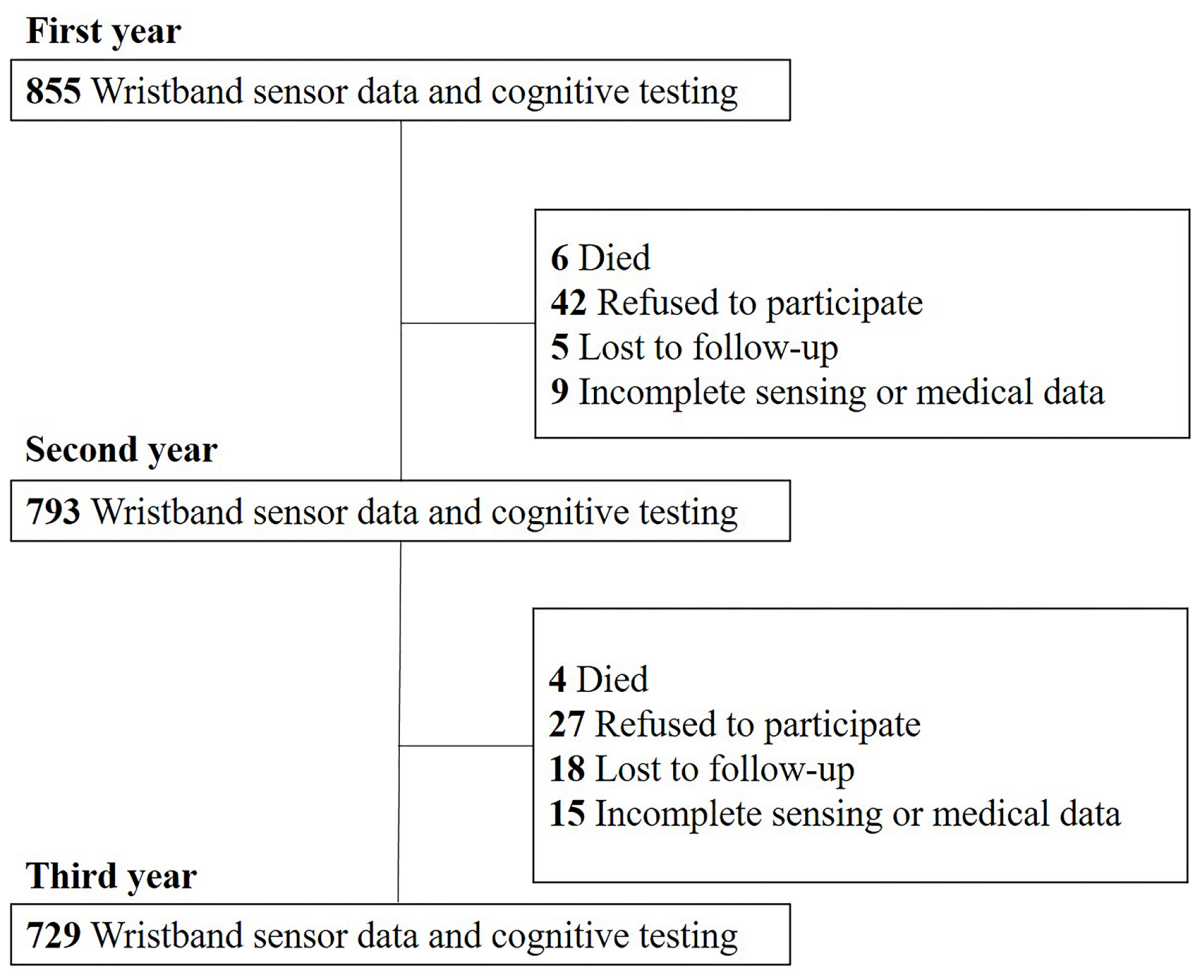
Flow of participant recruitment. 855 older adults who met the criteria had valid sensing data for analysis in the first year. The number of participants from whom valid wearable sensor data and cognitive testing results were completely collected during follow-up was 793 (92.7%) in the second year and 729 (85.3%) in the third year.

The Usuki study is a prospective cohort study of non-demented community-dwelling older adults conducted from August 2015 to September 2019 in Usuki, Oita Prefecture, Japan exploring risk and protective lifestyle factors for cognitive decline in later life (28, 29). The detailed design and methods have been described elsewhere (28). The inclusion criteria were as follows: (1) age ≥ 65 years; (2) residing in Usuki; (3) physically and psychologically healthy; (4) absence of dementia; and (5) independent function regarding activities of daily living. Participants who self-reported a dementia diagnosis or the use of dementia medication were excluded. This study was limited to adults aged 65 years and older because of their high risk of cognitive impairment or dementia and the target of the intervention. All participants were required to wear a wristband sensor (Silmee^™^ W20, TDK Corporation, Tokyo, Japan) continuously except when bathing for seven consecutive days every three months (four times per year) and to undergo annual cognitive assessments using the MMSE over a three-year follow-up period. Valid sensing data was defined as those obtained over least 3 days in one period and in at least two period per year as previously described (30). The mean [standard deviation (SD)] total measurement period was 89.2 (25.3) days. The MMSE results were reviewed by a neurologist and clinical psychologist for the primary screening for dementia. Demographic information, including age, sex, education level, body mass index, medication history, and dementia diagnosis or administration of dementia medication in the local hospital were obtained from the participants and their closest relatives via face-to-face clinical interviews by trained medical staff every year. At the baseline, 855 older adults {317 men (37.1%) and 538 women (62.9%), with a median age of 73 years [interquartile range (IQR): 69–78] and a median educational level of 12 years (IQR: 11–12)} who met the criteria had valid sensing data for analysis. The numbers of participants from whom valid wearable sensor data and cognitive testing results were completely collected during follow-up were 793 (92.7%) in second year (from 2016 to 2018) and 729 (85.3%) in the third year (from 2017 to 2019). Of the remaining 126 (14.8%) participants, 10 (1.2%) died, 69 (8.1%) refused to participate in this study, 23(2.7%) were lost to follow-up, and 24 (2.8%) had incomplete sensing or medical data (Figure 1). This prospective study was conducted in accordance with the Helsinki Declaration and was approved by the local ethics committee of Oita University Hospital (UMIN000017442). All participants provided written informed consent.

### Wearable sensor data

We excluded data indicating that the wristband sensor had been removed according to the heart rate. Variables were calculated by summing the sensor data collected on each day. Sleep–wake variables such as the total sleep time (TST), sleep efficiency, time awake after sleep onset (WASO), and waking frequency were measured during nighttime. The time of sleep onset was defined as the clock time when the first 20 min block of the resting state without movement began. Nocturnal waking was defined as 5–90 min of continuous movement during a continuous sleep period. Sleep efficiency was calculated as a percentage of TST versus the time spent in bed. WASO and waking frequency were calculated by averaging the total number of minutes awake after sleep onset and the number of awakenings per day, respectively. Naptime was defined as the resting period without movement on the wearable sensor during daytime. Physical activity data were detected by a three-axis accelerometer that measured acceleration in three perpendicular axes. Data were captured continuously and summarized in 1 min intervals. Steps were defined as walking in the frequency range of 2–3 Hz of acceleration as detected by the wristband sensor. Additionally, this device computed the intensity of activity as metabolic equivalents (METs). We classified physical activity intensity into three categories, namely sedentary behavior (≤1.5 METs), light physical activity (LPA; 1.6–2.9 METs), and moderate-to-vigorous physical activity (MVPA: ≥3.0 METs), as previously described (31). The absolute time spent in sedentary behavior, LPA, and MVPA was measured when participants were awake. We verified the measurement accuracy for walking steps and sleep time by comparing the sensor data with video observation data in healthy adults aged 20–80 years (28). Significant correlation was found between sleep duration and walking steps from wristband sensor and those from video observation (*r* = 0.9995, *r* = 0.9869, respectively, Pearson correlation).

### Statistical analysis

We first conducted principal component analysis (PCA) to reduce the dimensions of the various sleep and physical activity variables to two principal components for inclusion in a mixed-effects model. PCA was used to rank the physical activity and sleep variables by their relative importance and reduce the dimensionality of highly correlated original variables, including TST, sleep efficiency, WASO, waking frequency, and naptime, steps per day, LPA, MVPA, sedentary behavior at baseline. All 9 variables were subjected for PCA. Two main principal components emerged with eigenvalues >2. These components were extracted by contributing six variables with relatively high loadings to two components, such as the sleep and physical activity indices. Assuming the estimated component loadings were invariant to follow-up times, baseline scoring algorithm was applied to all follow-up variables to construct two time-varying. A linear mixed-effects model was used to examine whether the two time-varying indices were longitudinally associated with MMSE scores during follow-up after controlling age, sex, education level as potential confounders. The effect of follow-up time was modeled as discrete, and the interaction between two time-varying indices and follow-up time was not included in the model due to clinical and statistical difficulties in parameter interpretation. Compound-symmetry structure was specified for within-subject serial correlation among repeated-measures of MMSE by including random intercept term in the model. Statistical analyses were conducted using IBM SPSS Statistics version 25.0 (IBM Corp., Armonk, NY, United States) and JMP Pro 14.2.0 (SAS Institute Japan Ltd., Tokyo, Japan).

## Results

### Clinical and demographic characteristics

Table 1 summarizes the annual changes in demographic characteristics, MMSE scores, and wearable sensor data of all participants. At baseline, the mean (SD) daily TST was 410.7 min (68.6), the median (IQR) daily sleep efficiency was 96.4% (94.3–97.9), the median (IQR) daily WASO was 15.4 min (8.6–24.4), the median (IQR) waking frequency was 0.38 times/day (0.22–0.6), and the median (IQR) naptime was 34.8 min/day (18.7–61.9 min). The median (IQR) steps per day was 5,113 (3337.4–7093.5), the median (IQR) times devoted to LPA, MVPA, and sedentary behavior were 21.1 (11.9–33.7), 23.6 (14.1–37.1), and 782 min/day (733.1–821.7), respectively. The median (IQR) MMSE score was 29 points (27–30) at baseline.

**Table 1:** Clinical and demographic characteristics of all participants.

Characteristics	First year (*n* = 855)	Second year (*n* = 793)	Third year (*n* = 729)
Age, median (IQR), years	73 (69–78)	74 (70–78)	75 (71–79)
Sex (M:W)	317:538	289:504	263:466
Educational level, median (IQR), years	12 (11–12)	12 (11–12)	12 (11–12)
BMI, median (IQR), kg/m^2^	23 (21.1–25.1)	23 (21.2–25.1)	23.2 (21.4–25.2)
MMSE, median (IQR), score	29 (27–30)	29 (28–30)	29 (28–30)
TST, mean (SD), min/day	410.7 (68.6)	410.1 (70)	411.7 (70)
WASO, median (IQR), min/day	15.4 (8.6–24.4)	15.9 (8.6–24.9)	16 (8.2–25.7)
Sleep efficiency, median (IQR), %/day	96.4 (94.3–97.9)	96.2 (94.2–98)	96.4 (94–98)
Waking frequency median (IQR), times/day	0.38 (0.22–0.6)	0.4 (0.22–0.6)	0.38 (0.21–0.63)
Naptime, median (IQR), min/day	34.8 (18.7–61.9)	35.3 (18.7–61.4)	35.6 (18.9–64.8)
Walking steps, median (IQR), steps/day	5,113 (3,337.4–7,093.5)	5,022.8 (3,175.2–6,910.5)	4,706 (3,123.2–6,900.7)
LPA, median (IQR), min/day	21.1 (11.9–33.7)	21.5 (12–32.7)	19.6 (11–31.9)
MVPA, median (IQR), min/day	23.6 (14.1–37.1)	22.3 (12.8–35.1)	21.2 (12.2–33.8)
Sedentary, median (IQR), min/day	782 (733.1–821.7)	780.4 (733.1–824.2)	783.1 (731.7–825.8)

IQR, interquartile range; M, man; W, woman; BMI, body mass index; MMSE, mini-mental state examination; SD, standard deviation; min, minute; TST, total sleep time; WASO, time awake after sleep; LPA, light physical activity; MVPA, moderate-to-vigorous physical activity.

### Principal component analysis

**Figure 2:**
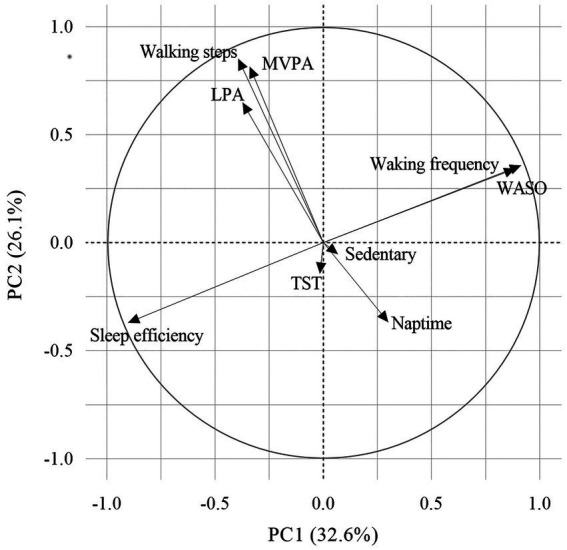
Principal component analysis of the variables. Principal component analysis reduced the dimensions of the various lifestyle factors to two principal components. The direction and relative importance of each variable are indicated by arrows. PCs 1 and 2 explained 32.6% and 26.1% of the total data variance, respectively. LPA, light physical activity; MVPA, moderate-to-vigorous physical activity; PC, principal component; TST, total sleep time; and WASO, time awake after sleep.

The sleep and physical activity indices were derived from wearable sensor data using principal component analysis. The first principal component termed the “sleep index” was mainly dominated by sleep efficiency, WASO, and waking frequency, whereas the second principal component termed the “physical activity index” was mainly dominated by steps per day, LPA, and MVPA (Figure 2). Sleep efficiency moved in the opposite direction of WASO and waking frequency, whereas steps per day, LPA, and MVPA moved in the same direction.

The sleep index was calculated as follows:


$$\begin{array}{c} Sleepindex=\left(0.048977\right)\times WASO+\left(-0.200036\right)\\ \times sleepefficiency+\left(2.045641\right)\\ \times wakingfrequency+17.582924 \end{array}$$

The physical activity index was calculated as follows:


$$\begin{array}{c} Physicalactivityindex=\left(0.000223\right)\times walkingsteps\\ +\left(0.029074\right)\times LPA+\left(0.033679\right)\\ \times MVPA+\left(-3.043219\right) \end{array}$$

Therefore, a higher sleep index indicated poor sleep quality, whereas a lower physical activity index indicated less physical activity. These two principal components that accounted for 58.7% of the total variance of the data.

### Association of time-specific changes in physical activity and sleep variables with those in MMSE score

Table 2 presents the results of the linear mixed-effects models estimating the longitudinal associations of the physical activity index, sleep index, and demographic factors with MMSE scores. After adjusting for age, sex, education level, and time, time-specific changes in the physical activity index were positively associated with MMSE scores (estimate, 0.06699; standard error, 0.03343; *p* = 0.0453), whereas those of the sleep index were inversely associated with MMSE scores (estimate, −0.06229; standard error, 0.02202; *p* = 0.0047).

**Table 2:** Linear mixed-effects models estimating the longitudinal associations of the sleep index and physical activity index with MMSE scores.

Variables	Estimate	SE	*p-*value
Sleep index	−0.06229	0.02202	0.0047*
Physical activity index	0.06699	0.03343	0.0453*
Year (1)	−0.364	0.06652	<0.0001*
Year (2)	−0.1791	0.06542	0.0063*
Age	−0.08481	0.009933	<0.0001*
Sex	0.2284	0.108	0.0347*
Education	0.1467	0.0258	<0.0001*

After adjusting for age, sex, educational level, and time, the sleep index was inversely associated with MMSE scores (estimate, −0.06229; standard error, 0.02202; *p* = 0.0047), whereas the physical activity index was positively associated with MMSE scores (estimate, 0.06699; standard error, 0.03343; *p* = 0.0453). SE, Standard error. * *p* < 0.05.

## Discussion

In this prospective cohort study, we identified longitudinal associations of objectively and simultaneously measured sleep and physical activity with MMSE score over a three-year follow-up period in community-dwelling older adults. Our findings indicated that poor sleep quality and lower level of physical activity were associated with cognitive decline. The present study findings contribute to the development of novel evidence-based interventions for sleep quality and physical activity in older adults to delay cognitive decline.

The most interesting finding of the present study was the longitudinal association of objectively measured sleep quality with cognitive decline. Sleep is important for memory consolidation and health-related quality of life. Sleep disturbances affect up to 50% of community-dwelling older adults and are bi-directionally linked to an increased risk of cognitive decline, or AD (4, 5). Previous population-based prospective studies commonly demonstrated that baseline levels of self-reported or objectively measured sleep variables, such as abnormal sleep duration, lower sleep efficiency, higher WASO or waking frequency, poor sleep quality, and longer sleep latency were associated with an increased risk of cognitive decline or dementia in cognitively healthy older adults (10–13). However, another study showed no significant association between self-reported sleep patterns and cognitive decline in older women (32). On the other hand, only a few studies have examined the longitudinal association between self-reported sleep variables and cognitive decline (22–24). These studies found an association between chronic insomnia and adverse changes in sleep duration (decreased from 6–8 h or increased from 7 or 8 h) and an increased risk of cognitive decline over 3–5 years of follow-up. Our results suggested that maintaining sleep quality with age was an important determinant in preventing dementia. The potential mechanisms linking sleep and cognitive function have been identified in human and animal models of AD (33–35). The physiological fluctuations in amyloid β (Aβ) are disrupted in AD mouse models and the older adults with an amyloid burden on PET imaging (33, 34). Furthermore, sleep deprivation acutely increased soluble Aβ levels in the interstitial fluid and chronically developed amyloid plaque formation in a mouse model of AD (34) and one night of sleep deprivation is negatively associated with amyloid burden in the human brain (35). These results suggested that chronic sleep deprivation increased Aβ production and reduce Aβ clearance, leading to amyloid plaque formation.

Another interesting finding of the present study was the longitudinal association of physical activity index with MMSE score over time. Physical activity is an important protective factor for age-related cognitive decline. Previous population-based prospective studies consistently found that lower levels of self-reported or objectively measured physical activity, such as total daily physical activity, total energy expenditure, low-to-moderate or vigorous physical activity, and walking, at baseline were associated with cognitive decline or an increased risk of AD in older adults (14–18). To the best of our knowledge, only a few studies have examined the dynamic association between self-reported physical activity and cognitive function (25–27). These studies found that physical activity duration or intensity were longitudinally associated with cognitive decline over a 10-year follow-up period. The present study is the first to confirm the longitudinal association between objectively measured physical activity and cognitive function in older adults. These findings suggest that maintaining physical activity with age is an important determinant in preventing dementia. Several possible mechanisms underlying the protective effect of physical activity on cognitive function have been proposed on the basis of human and animal studies (36–41). Physical activity may increase cerebral blood flow by reducing vascular risk factors (36), directly promoting neurogenesis (37), angiogenesis (38), synaptic plasticity, and stimulating neurotrophic factors (39). Moreover, physical activity was associated with a decreased Aβ burden on positron emission tomography imaging and higher Aβ42 levels in the cerebrospinal fluid of non-demented older adults as well as decreased amyloid plaques in a transgenic mouse model (40, 41).

This study had several strengths, including a large and prospective population-based cohort, objective measurements of lifestyle factor every three months and assessment of the MMSE every year over three years of follow-up. However, several limitations must be considered. First, the possibility of reverse causation could not be ruled out due to the relatively short follow-up period. Although we collected clinical information to define the presence or absence of dementia at baseline, patients with preclinical dementia could not be completely excluded from participating in the current study. Second, the 126 participants who were excluded from the analysis were older and had lower MMSE scores and physical activity levels than those who completed the follow-up (Supplementary Table S1). Therefore, excluding these participants may cause distortions in the results. Third, the use of the MMSE, which is widely used to screen patients with cognitive decline has limitations due to ceiling effects in community-dwelling cohorts.

## Conclusion

We confirmed that poor sleep quality and lower levels of physical activity were associated with subsequent cognitive decline over 3 years of follow-up in community-dwelling older adults. As sleep disturbance and physical inactivity are major problems in an aging society, we recommended intervention for improving these variables to prevent dementia or AD in older adults.

## Data availability statement

The original contributions presented in the study are included in the article/Supplementary material, further inquiries can be directed to the corresponding author.

## Ethics statement

The studies involving human participants were reviewed and approved by local ethics committee of Oita University Hospital (UMIN000017442). The patients/participants provided their written informed consent to participate in this study.

## Author contributions

NK and EM conceived and designed the trial. NK, TM, TA, and EM performed the acquisition or interpretation of data. YS and TK developed analysis plan and conducted statistical analysis. NK and TK drafted the original manuscript. AE performed the neuropsychological assessment. All authors contributed to the article and approved the submitted version.

## Funding

This research was supported by Japan Agency for Medical Research and Development (grant number 18he142003h003) and Grant-in-Aid for Scientific Research (C) under Grant Number 22K07474.

## Conflict of interest

NK received grant from Japan Society for the Promotion of Science; and honorarium from Takeda Pharmaceutical, Daiichi Sankyo, Eisai, Sumitomo Pharma, Kyowa Kirin, and Otsuka Pharmaceutical outside the submitted work. No other disclosures were reported.

The remaining authors declare that the research was conducted in the absence of any commercial or financial relationships that could be construed as a potential conflict of interest.

## Publisher’s note

All claims expressed in this article are solely those of the authors and do not necessarily represent those of their affiliated organizations, or those of the publisher, the editors and the reviewers. Any product that may be evaluated in this article, or claim that may be made by its manufacturer, is not guaranteed or endorsed by the publisher.

## References

[1] Kasajima M Eggleston K Kusaka S Matsui H Tanaka T Son BK. Projecting prevalence of frailty and dementia and the economic cost of care in Japan from 2016 to 2043: a microsimulation modelling study. Lancet Public Health. (2022) 7:e458–68. doi: 10.1016/S2468-2667(22)00044-5, PMID: 35487231 PMC9094718

[2] Livingston G Sommerlad A Orgeta V Costafreda SG Huntley J Ames D. Dementia prevention, intervention, and care. Lancet. (2017) 390:2673–734. doi: 10.1016/S0140-6736(17)31363-6 28735855

[3] Fratiglioni L Paillard-Borg S Winblad B. An active and socially integrated lifestyle in late life might protect against dementia. Lancet Neurol. (2004) 3:343–53. doi: 10.1016/S1474-4422(04)00767-7, PMID: 15157849

[4] Foley DJ Monjan AA Brown SL Simonsick EM Wallace RB Blazer DG. Sleep complaints among elderly persons: an epidemiologic study of three communities. Sleep. (1995) 18:425–32. doi: 10.1093/sleep/18.6.425 7481413

[5] Ju YE Lucey BP Holtzman DM. Sleep and Alzheimer disease pathology—a bidirectional relationship. Nat Rev Neurol. (2014) 10:115–9. doi: 10.1038/nrneurol.2013.269, PMID: 24366271 PMC3979317

[6] Lo JC Groeger JA Cheng GH Dijk DJ Chee MW. Self-reported sleep duration and cognitive performance in older adults: a systematic review and meta-analysis. Sleep Med. (2016) 17:87–98. doi: 10.1016/j.sleep.2015.08.021, PMID: 26847980

[7] Sofi F Valecchi D Bacci D Abbate R Gensini GF Casini A. Physical activity and risk of cognitive decline: a meta-analysis of prospective studies. J Intern Med. (2011) 269:107–17. doi: 10.1111/j.1365-2796.2010.02281.x 20831630

[8] Miner B Stone KL Zeitzer JM Han L Doyle M Blackwell T. Self-reported and actigraphic short sleep duration in older adults. J Clin Sleep Med. (2022) 18:403–13. doi: 10.5664/jcsm.9584, PMID: 34338629 PMC8804982

[9] VandeBunte A Gontrum E Goldberger L Fonseca C Djukic N You M. Physical activity measurement in older adults: wearables versus self-report. Front Digit Health. (2022) 4:869790. doi: 10.3389/fdgth.2022.869790, PMID: 36120711 PMC9470756

[10] Blackwell T Yaffe K Laffan A Ancoli-Israel S Redline S Ensrud KE. Associations of objectively and subjectively measured sleep quality with subsequent cognitive decline in older community-dwelling men: the MrOS sleep study. Sleep. (2014) 37:655–63. doi: 10.5665/sleep.3562, PMID: 24899757 PMC4044750

[11] Lim AS Kowgier M Yu L Buchman AS Bennett DA. Sleep fragmentation and the risk of incident Alzheimer’s disease and cognitive decline in older persons. Sleep. (2013) 36:1027–32. doi: 10.5665/sleep.2802, PMID: 23814339 PMC3669060

[12] Diem SJ Blackwell TL Stone KL Yaffe K Tranah G Cauley JA. Measures of sleep-wake patterns and risk of mild cognitive impairment or dementia in older women. Am J Geriatr Psychiatry. (2016) 24:248–58. doi: 10.1016/j.jagp.2015.12.002, PMID: 26964485 PMC4807599

[13] Chen JC Espeland MA Brunner RL Lovato LC Wallace RB Leng X. Sleep duration, cognitive decline, and dementia risk in older women. Alzheimers Dement. (2016) 12:21–33. doi: 10.1016/j.jalz.2015.03.004, PMID: 26086180 PMC4679723

[14] Yaffe K Barnes D Nevitt M Lui LY Covinsky K. A prospective study of physical activity and cognitive decline in elderly women: women who walk. Arch Intern Med. (2001) 161:1703–8. doi: 10.1001/archinte.161.14.1703 11485502

[15] Abbott RD White LR Ross GW Masaki KH Curb JD Petrovitch H. Walking and dementia in physically capable elderly men. JAMA. (2004) 292:1447–53. doi: 10.1001/jama.292.12.1447, PMID: 15383515

[16] Buchman AS Boyle PA Yu L Shah RC Wilson RS Bennett DA. Total daily physical activity and the risk of AD and cognitive decline in older adults. Neurology. (2012) 78:1323–9. doi: 10.1212/WNL.0b013e3182535d35, PMID: 22517108 PMC3335448

[17] Middleton LE Manini TM Simonsick EM Harris TB Barnes DE Tylavsky F. Activity energy expenditure and incident cognitive impairment in older adults. Arch Intern Med. (2011) 171:1251–7. doi: 10.1001/archinternmed.2011.277, PMID: 21771893 PMC3923462

[18] Stubbs B Chen LJ Chang CY Sun WJ Ku PW. Accelerometer-assessed light physical activity is protective of future cognitive ability: a longitudinal study among community dwelling older adults. Exp Gerontol. (2017) 91:104–9. doi: 10.1016/j.exger.2017.03.003 28263868

[19] Lohne-Seiler H Hansen BH Kolle E Anderssen SA. Accelerometer-determined physical activity and self-reported health in a population of older adults (65–85 years): a cross-sectional study. BMC Public Health. (2014) 14:284. doi: 10.1186/1471-2458-14-284, PMID: 24673834 PMC3984636

[20] Ohayon MM. Epidemiology of insomnia: what we know and what we still need to learn. Sleep Med Rev. (2002) 6:97–111. doi: 10.1053/smrv.2002.0186, PMID: 12531146

[21] Butler SM Ashford JW Snowdon DA. Age, education, and changes in the Mini-mental state exam scores of older women: findings from the Nun study. J Am Geriatr Soc. (1996) 44:675–81. doi: 10.1111/j.1532-5415.1996.tb01831.x, PMID: 8642159

[22] Cricco M Simonsick EM Foley DJ. The impact of insomnia on cognitive functioning in older adults. J Am Geriatr Soc. (2001) 49:1185–9. doi: 10.1046/j.1532-5415.2001.49235.x 11559377

[23] Ferrie JE Shipley MJ Akbaraly TN Marmot MG Kivimäki M Singh-Manoux A. Change in sleep duration and cognitive function: findings from the Whitehall II study. Sleep. (2011) 34:565–73. doi: 10.1093/sleep/34.5.565, PMID: 21532949 PMC3079935

[24] Loerbroks A Debling D Amelang M Stürmer T. Nocturnal sleep duration and cognitive impairment in a population-based study of older adults. Int J Geriatr Psychiatry. (2010) 25:n/a–109. doi: 10.1002/gps.2305 19548221

[25] van Gelder BM Tijhuis MA Kalmijn S Giampaoli S Nissinen A Kromhout D. Physical activity in relation to cognitive decline in elderly men: the FINE study. Neurology. (2004) 63:2316–21. doi: 10.1212/01.wnl.0000147474.29994.35 15623693

[26] Ku PW Stevinson C Chen LJ. Prospective associations between leisure-time physical activity and cognitive performance among older adults across an 11-year period. J Epidemiol. (2012) 22:230–7. doi: 10.2188/jea.je20110084, PMID: 22343329 PMC3798624

[27] Lindwall M Cimino CR Gibbons LE Mitchell MB Benitez A Brown CL. Dynamic associations of change in physical activity and change in cognitive function: coordinated analyses of four longitudinal studies. J Aging Res. (2012) 2012:493598. doi: 10.1155/2012/493598, PMID: 23029615 PMC3457643

[28] Kimura N Aso Y Yabuuchi K Ishibashi M Hori D Sasaki Y. Modifiable lifestyle factors and cognitive function in older people: a cross-sectional observational study. Front Neurol. (2019) 10:401. doi: 10.3389/fneur.2019.00401, PMID: 31068892 PMC6491512

[29] Kimura N Aso Y Yabuuchi K Ishibashi M Hori D Sasaki Y. Association of modifiable lifestyle factors with cortical amyloid burden and cerebral glucose metabolism in older adults with mild cognitive impairment. JAMA Netw Open. (2020) 3:e205719. doi: 10.1001/jamanetworkopen.2020.5719, PMID: 32515796 PMC7284299

[30] Barnes DE Blackwell T Stone KL Goldman SE Hillier T Yaffe K. Cognition in older women: the importance of daytime movement. J Am Geriatr Soc. (2008) 56:1658–64. doi: 10.1111/j.1532-5415.2008.01841.x, PMID: 18662201 PMC2680379

[31] Pate RR Pratt M Blair SN Haskell WL Macera CA Bouchard C. Physical activity and public health. A recommendation from the Centers for Disease Control and Prevention and the American College of Sports Medicine. JAMA. (1995) 273:402–7. doi: 10.1001/jama.273.5.402, PMID: 7823386

[32] Tworoger SS Lee S Schernhammer ES Grodstein F. The association of self-reported sleep duration, difficulty sleeping, and snoring with cognitive function in older women. Alzheimer Dis Assoc Disord. (2006) 20:41–8. doi: 10.1097/01.wad.0000201850.52707.80 16493235

[33] Roh JH Huang Y Bero AW Kasten T Stewart FR Bateman RJ. Disruption of the sleep-wake cycle and diurnal fluctuation of β-amyloid in mice with Alzheimer’s disease pathology. Sci Transl Med. (2012) 4:150ra122. doi: 10.1126/scitranslmed.3004291, PMID: 22956200 PMC3654377

[34] Kang JE Lim MM Bateman RJ Lee JJ Smyth LP Cirrito JR. Amyloid-beta dynamics are regulated by orexin and the sleep-wake cycle. Science. (2009) 326:1005–7. doi: 10.1126/science.1180962, PMID: 19779148 PMC2789838

[35] Shokri-Kojori E Wang GJ Wiers CE Demiral SB Guo M Kim SW. β-Amyloid accumulation in the human brain after one night of sleep deprivation. Proc Natl Acad Sci U S A. (2018) 115:4483–8. doi: 10.1073/pnas.1721694115, PMID: 29632177 PMC5924922

[36] Rogers RL Meyer JS Mortel KF. After reaching retirement age physical activity sustains cerebral perfusion and cognition. J Am Geriatr Soc. (1990) 38:123–8. doi: 10.1111/j.1532-5415.1990.tb03472.x, PMID: 2299115

[37] van Praag H Kempermann G Gage FH. Running increases cell proliferation and neurogenesis in the adult mouse dentate gyrus. Nat Neurosci. (1999) 2:266–70. doi: 10.1038/6368, PMID: 10195220

[38] Black JE Isaacs KR Anderson BJ Alcantara AA Greenough WT. Learning causes synaptogenesis, whereas motor activity causes angiogenesis, in cerebellar cortex of adult rats. Proc Natl Acad Sci U S A. (1990) 87:5568–72. doi: 10.1073/pnas.87.14.5568, PMID: 1695380 PMC54366

[39] Cotman CW Berchtold NC. Exercise: a behavioral intervention to enhance brain health and plasticity. Trends Neurosci. (2002) 25:295–301. doi: 10.1016/s0166-2236(02)02143-4, PMID: 12086747

[40] Liang KY Mintun MA Fagan AM Goate AM Bugg JM Holtzman DM. Exercise and Alzheimer’s disease biomarkers in cognitively normal older adults. Ann Neurol. (2010) 68:311–8. doi: 10.1002/ana.22096, PMID: 20818789 PMC2936720

[41] Adlard PA Perreau VM Pop V Cotman CW. Voluntary exercise decreases amyloid load in a transgenic model of Alzheimer’s disease. J Neurosci. (2005) 25:4217–21. doi: 10.1523/JNEUROSCI.0496-05.2005, PMID: 15858047 PMC6725122

