# Supplementary material for: Lifestyle factors that affect cognitive function–a longitudinal objective analysis
Source: Front Public Health. 2023 Jul 31;11:1215419. doi: 10.3389/fpubh.2023.1215419 (PMC10425549; doi:10.3389/fpubh.2023.1215419)
Supplement: Supplementary file 1 [file Table_1.docx]

**Supplemental table 1. Comparison between participants who completed and withdrew from the study.**

| **Characteristics** | **Completed (*n* = 729)** | **Withdrew (*n* = 126)** | ***p*-value** |
| --- | --- | --- | --- |
| Age, median (IQR), years | 73 (69–77) | 76 (72–81) | <0.001* |
| Sex (M:W) | 263:466 | 54:72 | 0.146 |
| Educational level, median (IQR), years | 12 (11–12) | 12 (9.75–12) | 0.349 |
| BMI, median (IQR), kg/m^2^ | 23 (21.2–25) | 23 (20–25.4) | 0.266 |
| MMSE, median (IQR), score | 29 (28–30) | 28 (26–30) | <0.001* |
| TST, mean (SD), min/day | 408.7 (66.4) | 422.1 (79.6) | 0.073 |
| WASO, median (IQR), min/day | 15.3 (8.6–24) | 16.2 (9.2–26) | 0.458 |
| Sleep efficiency, median (IQR), %/day | 96.4 (94.3–98) | 96.5 (93.8–97.9) | 0.800 |
| Waking frequency, median (IQR), times/day | 0.38 (0.22–0.58) | 0.38 (0.25–0.67) | 0.233 |
| Naptime, median (IQR), min/day | 34.2 (18.7–59.1) | 37.6 (18.3–83.4) | 0.123 |
| Walking steps, median (IQR), steps/day | 5254 (3454.7–7258.9) | 4174.7 (2440.3–6173.2) | <0.001* |
| LPA, median (IQR), min/day | 21.9 (12.5–34) | 15.8 (7.8–30.5) | <0.001* |
| MVPA, median (IQR), min/day | 24.5 (14.7–38.6) | 18.6 (9.7–28) | <0.001* |
| Sedentary, median (IQR), min/day | 784.9 (735.2–822.2) | 769.7 (695.4–809.2) | 0.007* |
| Conversation time, median (IQR), min/day | 224.7 (168.7–286.3) | 231.8 (158.1–317.5) | 0.576 |
| Heart rate, median (IQR), beats/min | 64.1 (60.6–68.5) | 63.8 (59.4–68.9) | 0.878 |

IQR, interquartile range; M, man; W, woman; BMI, body mass index; MMSE, Mini-Mental State Examination; SD, standard deviation; min, minute; TST, total sleep time; WASO, time awake after sleep; LPA, light physical activity; MVPA, moderate-to-vigorous physical activity.
